# Combined application of biochar and peatmoss for mitigation of drought stress in tobacco

**DOI:** 10.1186/s12870-024-05576-6

**Published:** 2024-09-14

**Authors:** Qamar uz Zaman, Muzammal Rehman, Youhong Feng, Zhiyuan Liu, Ghulam Murtaza, Khawar Sultan, Kamran Ashraf, Mohamed S. Elshikh, Dunia A. Al Farraj, Muhammad Rizwan, Rashid Iqbal, Gang Deng

**Affiliations:** 1 School of Agriculture, Yunnan University, Kunming, Yunnan 650504 China; 2 Department of Environmental Sciences, The University of Lahore, Lahore, 54590 Pakistan; 3 College of Agriculture, Guangxi Key Laboratory of Agro-Environment and Agric- Products Safety, Key Laboratory of Crop Genetic Breeding and Germplasm Innovation, Guangxi University, Nanning, 530004 China; 4 State Key Laboratory of Bioreactor Engineering, East China University of Science and Technology, Shanghai, 200237 PR China; 5 Department of Botany and Microbiology, College of Science, King Saud University, P.O. 2455, Riyadh, 11451 Saudi Arabia; 6 Institute of Crop Science and Resource Conservation (INRES), University of Bonn, 53115 Bonn, Germany; 7 Department of Agronomy, Faculty of Agriculture and Environment, The Islamia University of Bahawalpur, Bahawalpur, 63100 Pakistan; 8 Department of Life Sciences, Western Caspian University, Baku, Azerbaijan

**Keywords:** Biomass, Biochar, Drought, Growth, Peatmoss, Quality, Well-watered, Sustainable

## Abstract

Drought poses a significant ecological threat that limits the production of crops worldwide. The objective of this study to examine the impact of soil applied biochar (BC) and peatmoss (PM) on the morpho-biochemical and quality traits of tobacco plants under drought conditions. In the present experiment work, a pot trial was conducted with two levels of drought severity (~ well-watered 75 ± 5% field capacity) and severe drought stress (~ 35 ± 5% field capacity), two levels of peatmoss (PM) @ 5% [PM+ (with peatmoss) and PM- (without peatmoss)] and three levels of rice straw biochar (BC_0_ = no biochar; BC_1_ = 150 mg kg^− 1^; and BC_2_ = 300 mg kg^− 1^ of soil) in tobacco plants. The results indicate that drought conditions significantly impacted the performance of tobacco plants. However, the combined approach of BC and PM significantly improved the growth, biomass, and total chlorophyll content (27.94%) and carotenoids (32.00%) of tobacco. This study further revealed that the drought conditions decreased the production of lipid peroxidation and proline accumulation. But the synergistic approach of BC and PM application increased soluble sugars (17.63 and 12.20%), soluble protein (31.16 and 15.88%), decreased the proline accumulation (13.92 and 9.03%), and MDA content (16.40 and 8.62%) under control and drought stressed conditions, respectively. Furthermore, the combined approach of BC and PM also improved the leaf potassium content (19.02%) by limiting the chloride ions (33.33%) under drought stressed conditions. Altogether, the balanced application of PM and BC has significant potential as an effective approach and sustainable method to increase the tolerance of tobacco plants subjected to drought conditions. This research uniquely highlights the combined potential of PM and BC as an eco-friendly strategy to enhance plant resilience under drought conditions, offering new insights into sustainable agricultural practices.

## Introduction

A key challenge experienced by crops globally is the phenomenon of drought stress, which can greatly affect their growth and yield [1]. Unfortunately, the continual effects of global warming and climate change are projected to contribute to more frequent and severe drought conditions, resulting in a scarcity of water for agriculture [2]. Primary effects of drought stress on plants include disturbances in nutrient and water absorption, osmotic equilibrium, cellular growth, turgidity, and oxidative damage [3]. There was also a noticeable decrease in leaf area, photosynthetic pigments, and CO_2_ assimilation due to the drought stress [4]. Furthermore, when drought conditions take place, plants may experience stomatal closure, causing an increase in the production of reactive oxygen species (ROS). This excess of ROS can negatively impact the functioning of cells, leading to damage and ultimately cell death [5, 6].

Tobacco is of great economic importance and is widely grown in various locations, even in areas with varying soil and climate conditions [7]. The drought stress significantly affects essential physiological processes required for the growth and development of tobacco, therefore significantly limiting its yield [8]. During drought conditions, the decreased availability of water hinders transpiration, resulting in the closing of stomata to preserve water [9]. As result of the closure of stomatal pores, the uptake of CO₂ is restricted, leading to a reduction in photosynthetic rates and total biomass production. Furthermore, drought stress contributes to the accumulation of reactive oxygen species (ROS) levels and enzymatic and non-enzymatic antioxidants activities, leading to cellular and macromolecular damage [10]. The combined effects of oxidative stress and reduced synthesis of essential metabolites and proteins inhibit cell division and elongation, leading to reduced growth and lowered leaf yield and quality, ultimately decreasing tobacco productivity [11, 12]. Under drought conditions, the biomass of tobacco plants can decrease significantly, with studies reporting reductions of up to 40–60% in total dry matter [13]. Photosynthesis in tobacco under drought stress is often reduced by 30–50%, primarily due to stomatal closure, which limits carbon dioxide uptake [14]. Drought stress leads to a substantial decrease (40%) in the chlorophyll content of tobacco leaves, resulting in less efficient light capture and energy production [15]. Quantitative studies have shown that leaf yield can drop by as much as 50% under severe drought conditions [16].

Various methodologies, such as breeding technology, genetic engineering, and bioengineering, have been widely used to cultivate field crops conferred with drought tolerance [17]. However, due to their need for modern technology and significant time investment, these methods sometimes lack interest between the farming community [18]. Furthermore, a cost-efficient and easy approach to improve plant resilience to stress induced by drought conditions is to integrate soil amendments, including both organic and inorganic substances, into the soil [19]. Applying biochar (BC), a stable carbon-rich byproduct derived from biomass has shown great potential in enhancing soil quality and ultimately boosting crop productivity [20]. The use of biochar has been found to enhance crop productivity in drought stressed soils [21]. The application of BC has been found to enhance water use efficiency (WUE), nutrient uptake, carbon assimilation, and antioxidant activities, resulting in improved plant growth even in water deficit conditions [22]. It enhances chlorophyll synthesis, promotes stomata conductivity, helps maintain membrane stability, and prevents the overproduction of ROS [23]. These effects facilitate enhanced growth of plants, particularly in severe drought conditions. Furthermore, the incorporation of peatmoss into the soil can significantly enhance crop yields and provide economic advantages in regions with limited water resources [24]. It helps to reduce evapotranspiration and soil water consumption while increasing water use efficiency [25]. However, specific yield results may change among species of crops such as wheat, maize, and potato. In addition, peatmoss can improve soil quality by stimulating the proliferation of microorganisms and enhancing nitrogen availability [26]. This was particularly beneficial in mitigating oxidative damage caused by drought [27].

Previous studies have provided extensive knowledge on the impact of drought stress on tobacco productivity [28, 29]. However, specific physiological variables that cause a reduction in normal growth and quality of tobacco plants are still not well recognized. Moreover, there is a limited research on the combined effectiveness of integrating biochar and peatmoss as an effective way to enhance drought tolerance in tobacco plants. Having a deeper understanding of how the combined approach of biochars and peatmoss affects the physiological, antioxidative, osmolyte, and quality traits of tobacco under drought stress can greatly benefit sustainable agriculture. The objective of this work was to examine the synergistic impact of biochar and peatmoss integration on the growth, biomass, physiological and qualitative traits of tobacco under drought-induced stress conditions. The objective of our work was to evaluate the effects of biochar and peatmoss treatments on tobacco yield, individually and in combination. This study has examined three primary hypotheses: (1) Drought stress has the potential to reduce tobacco growth and biomass traits, leading to reduction in overall crop productivity. (2) The addition of biochar and peatmoss in individual and combined form has the ability to mitigate the negative effects of drought stress.

## Materials and methods

### Study plan

A pot trial experiment was conducted in the greenhouse of School of Agriculture, Yunnan University, Kunming, China from September 2023 to January 2024. In this study, the experimental treatments consisted of three factors, i.e., the drought stress (~ well-watered 75 ± 5% field capacity) and severe drought stress (~ 35 ± 5% field capacity); peatmoss (PM) @ 5% [PM+ (with peatmoss) and PM- (without peatmoss)] and various levels of rice straw biochar (BC_0_ = no biochar; BC_1_ = 150 mg kg^− 1^; and BC_2_ = 300 mg kg^− 1^ of soil). The experiment was designed by employing a completely randomized design (CRD) as a research tool under factorial arrangement and replicated thrice (each of the replications comprised 3 pots per treatment).

### Procurement of seeds and soil amendments

The tobacco cultivar used was Yunyan 87, obtained from the Yunnan Tobacco Company, Kunming, China. The rice straw biochar and peatmoss were purchased from the Yunnan Lvzhiyuan Fertilizer Co., Ltd. Kunming, China.

### Crop management

Soils with the loam texture, pH value of 5.11, EC of 175.43 mSm^− 1^, organic matter content of 18.22 g kg^− 1^, hydrolytic nitrogen concentration of 89.34 mg kg^1^, available phosphorus content of 82.34 mg kg^− 1^ and available potassium of 364.21 mg kg^− 1^ was chosen for this experiment work to fill the plastic pots. The diameter of the pots was about 40 cm the depth of 30 cm and were filled with about 10 kg of soil. The peatmoss and biochar were mixed in the soil as per the treatment plan and left for 15 days before the sowing of tobacco seeds for the complete homogenization of treatments with the soil. Manual hoeing was done on the basis of the homogenization of biochar and peatmoss with soil with a sprinkling of water on alternative days. Seed decontamination was done by using 0.1% (*w/v*) sodium dodecyl solution and then washing the seed samples with ultra-pure deionized water. The crop was supplemented with fertilizer to meet the nutritional requirements such as K_2_O: N: P_2_O_5_: 12.0,4.0, and 8.0 g pot^− 1^. For the irrigation requirement tap water was used to achieve and maintain the field capacity (FC) level every day throughout the experiment. Drought stress was imposed on tobacco plants after 50 days subsequent to the sowing process. Soil moisture was carefully monitored using an electronic balance after thinning the seedlings to effectively manage the impact of drought stress. Experimental pots were weighed and distilled water was used to replenish any water loss every 1 or 2 days, if required. The severity of drought stress was kept at the desired level by ensuring that the field capacity is maintained. By using the equation.

FC (%) = water added – water leached the field capacity (FC).

Throughout the experiment, the pots weighed when they were watered, and a sufficient amount of water was given to sustain the optimal soil moisture level at desired field capacity.This drought phase was sustained for a total duration of 20 days. Following the drought period, the plants were immediately rehydrated in water. Afterward, the irrigation and fertilization practices were resumed following the standard protocols for tobacco cultivation. Additional cultivation practices in Kunming, Yunnan Province, China were followed to meet the desired standards and improve the quality of tobacco.

### Data collection

#### Growth attributes

The growth and morphological traits such as plant height, stem diameter, leaf length, leaf width, leaf area and number of leaves of tobacco plants as per treatments were assessed following the guidelines outlined in the Investigating and Measuring Methods of Agronomical Character of Tobacco, as per the Tobacco Industry Standard of the People’s Republic of China YC/T142-2010. All growth attributes of the tobacco plants were measured and recorded at 90–100 days after sowing, as the plant has reached its final growth.

#### Biomass attributes

Following the harvest of tobacco plants, the roots were separated from the shoots, stems, and leaves. Following the measurement of the fresh biomass of roots and shoots using a weighing balance, the plant samples were dried for 48 h at 65℃ until their weight remained constant.

#### Photosynthetic attributes

From each treatment after 20 days of drought stress, young leaves were sampled in triplicates. After the process of crushing leaf samples in test tubes with 85% acetone (v/v) and allowing them to stand in the dark for 24 h was done to extract pigments. Subsequently, the sample was subjected to centrifugation at 4000 × g for 10 min at 4℃. Using a spectrophotometer (Halo DB-20/DB-20 S, UK), measurements of the supernatant were recorded at wavelengths of 470, 647, and 664.5 nm, following the procedure described by Lichtenthaler [30], in order to analyze the concentrations of chlorophyll a, chlorophyll b, and carotenoids. The total chlorophyll content was determined by adding up the concentrations of chlorophyll a and chlorophyll b.

#### Lipid per oxidation and enzymatic antioxidants attributes

The supernatant sample obtained from centrifuging a 1-gram leaf sample with a 50 mM phosphate buffer at 15,000 × g for 10 min was utilized to measure the activity of plant enzymes. The enzymatic activities (SOD, POD, and CAT), and lipid peroxidation attributes (MDA content) were determined using assay kits (A064, Nanjing Jiancheng Bioengineering Institute, Nanjing, China) following the procedures provided by the manufacturer. The spectrophotometric measurement of the decline in absorbance at wavelength 290 nm was used to determine the activity of ascorbic acid peroxidase (APX). One unit of APX was determined as the amount of enzyme required to oxidize 1 µmol of ascorbate per minute.

#### Osmolytes attributes

A fresh leaf sample weighing 0.5 g taken 20 days after drought stress was grounded with a buffer (pH 7.2). Protease inhibitors (1 µM) were added to a saline phosphate buffer. The pH of the saline buffer solution was adjusted using HCl, followed by autoclaving. The extract was then centrifuged at 12,000 × g for 5 min for the separation of supernatant. Proline content was determined following the method outlined by Chance and Maehly [31]. The soluble sugars and soluble protein contents were assessed using the techniques reported by Giannakoula et al. [32] and the Bradford assay [33] method, respectively.

#### Leaf quality attributes

After 20 days of drought stress, from each treatment, 2 g of leaves was sampled in triplicate form. Nicotine content was determined by reaction with sulphanilic acid and cyanogen chloride using protocols of Coresta [34]. For potassium (K) and chloride (Cl) concentration determination, the leaf samples were oven-dried, weighed, and ashed at 550 °C for 8 h in a muffle furnace. Flame photometry (PFP7, Jenway, UK) was utilized to determine K concentration, while chloride (Cl^−^) was determined by following the protocols of Gaines et al., [35].

#### Statistical analysis

A statistical analysis technique three-way ANOVA was performed on the dataset to investigate any significant differences and prevailing patterns among the treatments that were applied. Pearson’s correlation was used to explore the connections and associations between the variables. Data was analyzed using the statistical software package Statistics 8.01. The statistical and visualization tool of R-Studio software was used to compute principal component analysis, correlation, and chord analysis.

## Results

### Growth attributes

**Fig. 1:**
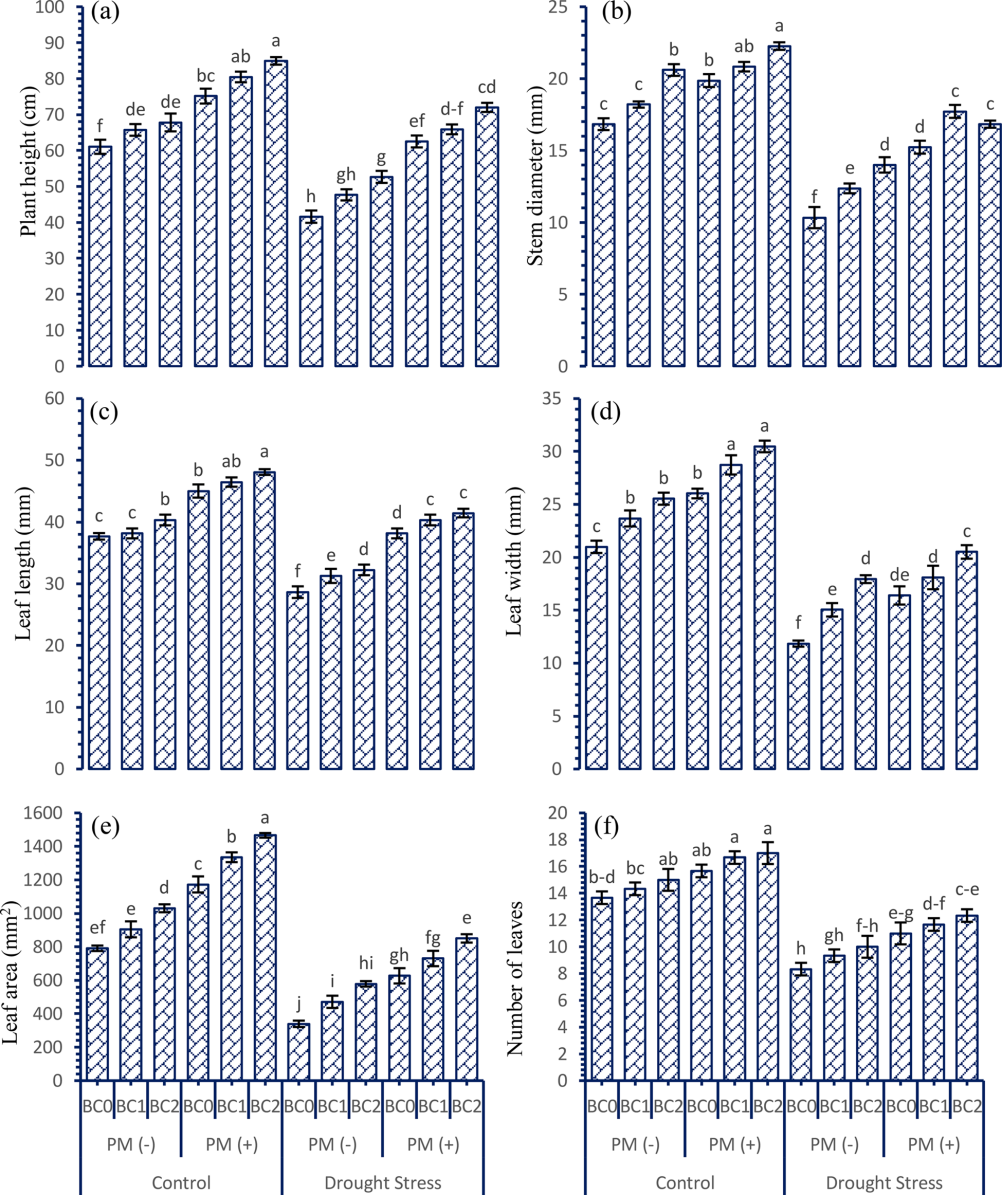
Interactive effect of various doses of soil applied biochar and peatmoss on the growth attributes of tobacco grown under drought stressed conditions. For each parameter, bars with the same letter are not significantly different across treatment means (*p* ≤ 0.05) based on a Tukey’s-HSD test. Capped lines denote the standard deviation of three replicates. PM (+); with peatmoss; PM (-); without peatmoss; BC_0_ = no biochar; BC_1_ = 150 mg kg^− 1^; BC_2_ = 300 mg kg^− 1^; **a**) plant height; **b**) stem diameter; **c**) leaf length; **d**) leaf width; **e**) leaf area; **f**) number of leaves

The findings of this study based on the data analysis showed that drought stress affected the growth attributes of tobacco plants. A linear decrease was noticed in all the growth attributes by the drought stress. However, the addition of peat moss (PM) and biochar (BC) at various levels significantly (*p* ≤ 0.05) contributed to the improvement of the growth traits of the tobacco plants under well-watered and drought-stressed conditions (Fig. 1a-f). Drought decreased the plant height (21.32%), leaf length (17.07%), leaf width (35.78%), leaf area (46.32%), stem diameter (27.07%), and leaf number (32.13%) as compared to control conditions. Application of BC (300 g kg^− 1^) and PM (5%) in the soil increased the plant height (12.17 and 19.76%), leaf length (6.01 and 10.32%), leaf width (19.14 and 36.03%), leaf area (27.21 and 47.82%), stem diameter (16.78 and 20.65%), and leaf number (9.06 and 15.51%) under control and drought stressed conditions. More response in terms of better growth attributes was noticed where BC was applied at the rate 300 g kg^− 1^ of soil combined with the peatmoss, while minimum response was observed where no BC and peatmoss was applied under drought stressed conditions.

### Biomass attributes

**Fig. 2:**
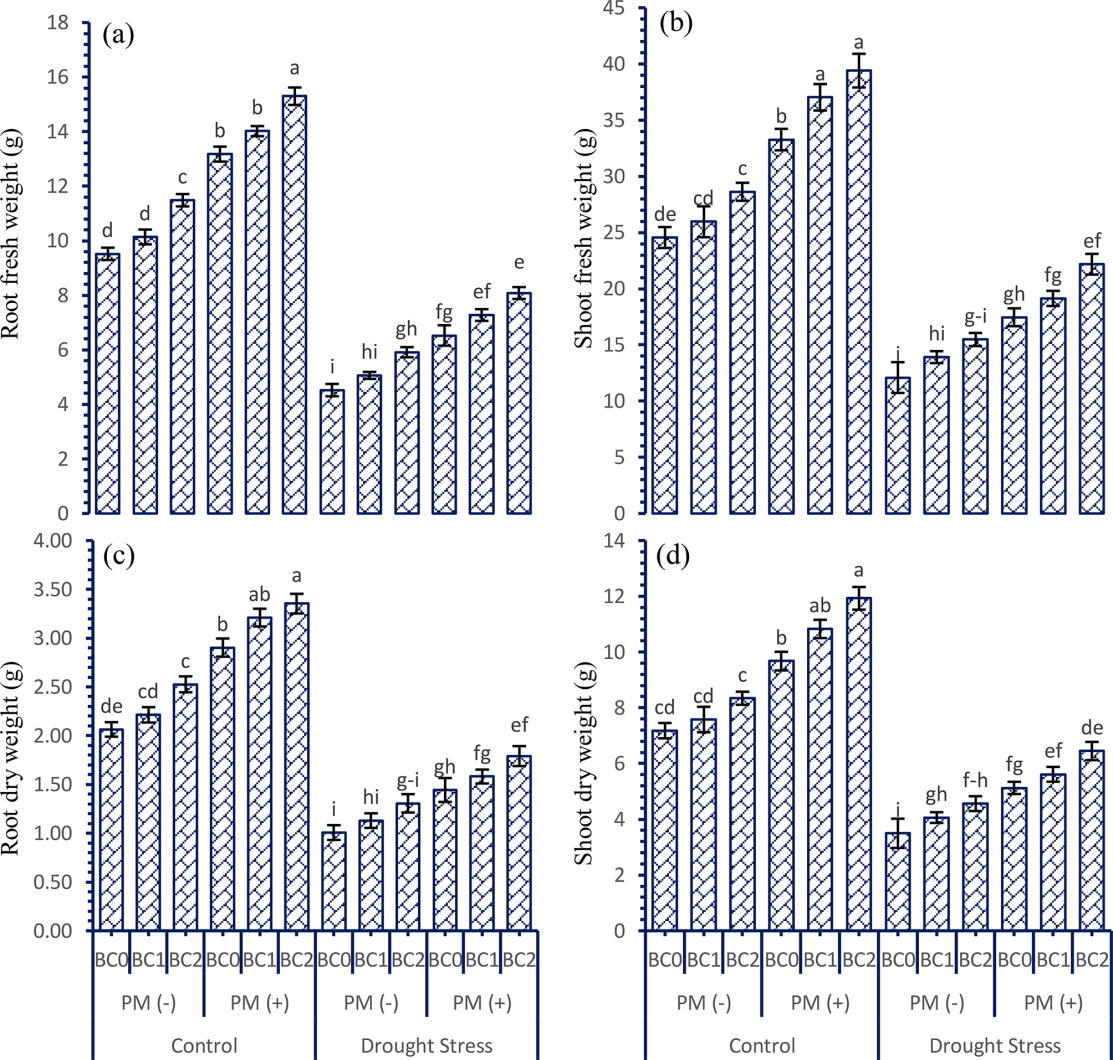
Interactive effect of various doses of soil applied biochar and peatmoss on the biomass (fresh and dry) attributes of tobacco grown under drought stressed conditions. For each parameter, bars with the same letter are not significantly different across treatment means (*p* ≤ 0.05) based on a Tukey’s-HSD test. Capped lines denote the standard deviation of three replicates. PM (+); with peatmoss; PM (-); without peatmoss; BC_0_ = no biochar; BC_1_ = 150 mg kg^− 1^; BC_2_ = 300 mg kg^− 1^; **a**) root fresh weight; **b**) shoot fresh weight; **c**) root dry weight; **d**) shoot dry weight

Data on biomass attributes of tobacco plants shown in Fig. 2a-d exhibited that drought stress decreased the biomass (fresh and dry) attributes. Drought conditions decreased the root fresh weight (49.23%), root dry weight (49.24%), shoot fresh weight (46.91%), and shoot dry weight (47.22%). However, the soil addition of BC and PM also improved the root fresh weight (17.97 and 26.58%), root dry weight (18.54 and 26.01%), shoot fresh weight (17.67 and 27.40%), and shoot dry weight (20.28 and 27.84%) in comparison with control where no BC and PM was applied under control and drought stressed conditions, respectively. The decreasing pattern for BC levels related to all the fresh and dry biomass attributes of tobacco plants was noted as 300 g kg^− 1^ > 150 g kg^− 1^ > control.

### Photosynthetic attributes

**Fig. 3:**
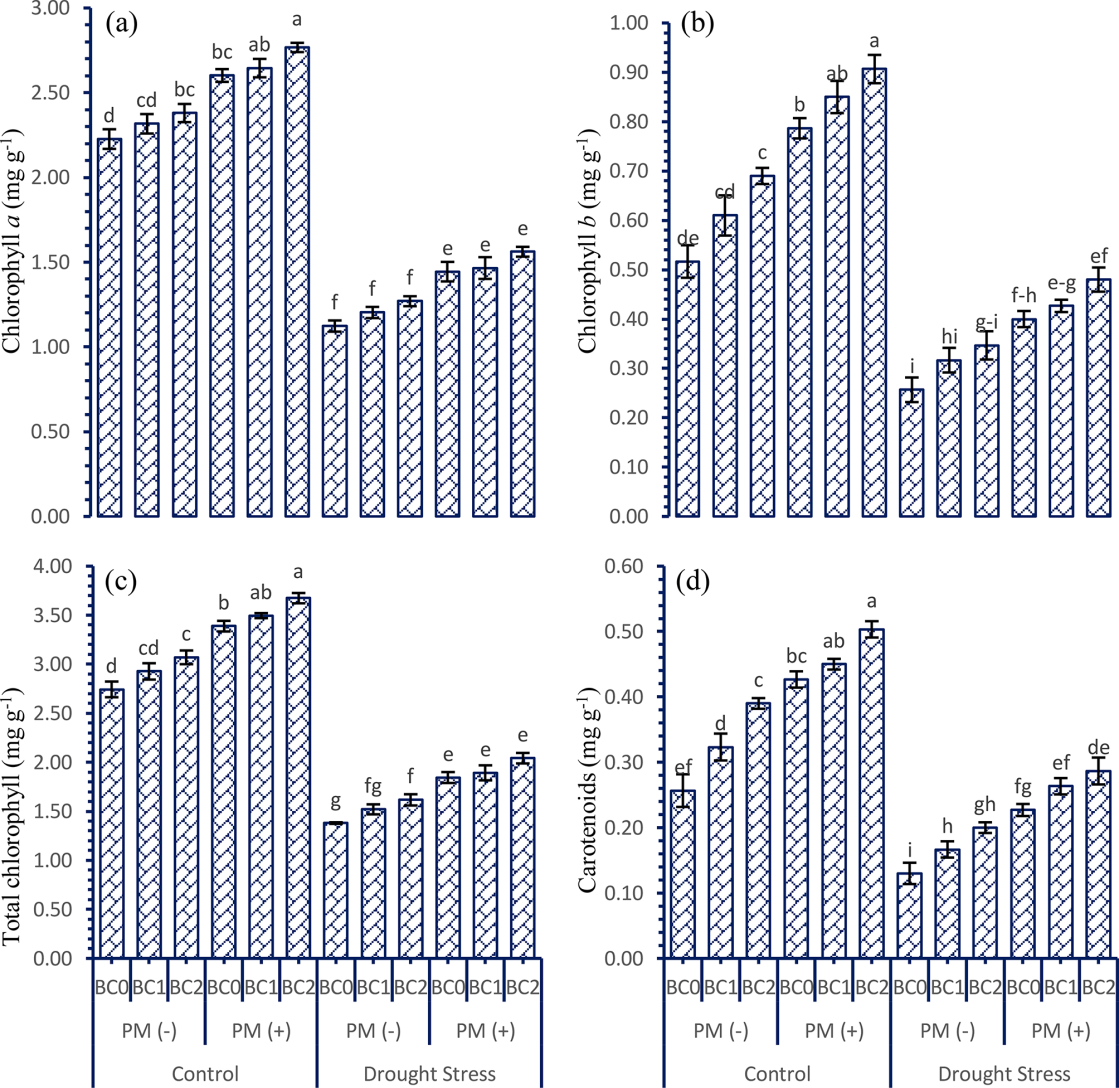
Interactive effect of various doses of soil applied biochar and peatmoss on the photosynthetic attributes of tobacco grown under drought stressed conditions. For each parameter, bars with the same letter are not significantly different across treatment means (*p* ≤ 0.05) based on a Tukey’s-HSD test. Capped lines denote the standard deviation of three replicates. PM (+); with peatmoss; PM (-); without peatmoss; BC_0_ = no biochar; BC_1_ = 150 mg kg^− 1^; BC_2_ = 300 mg kg^− 1^; **a**) chlorophyll a; **b**) chlorophyll b; **c**) total chlorophyll content; **d**) carotenoids content

Various levels of soil applied biochar in combination with and without peatmoss significantly improved the photosynthetic attributes of tobacco in comparison with control under the normal and drought stressed conditions (Fig. 3a-d). Drought conditions proved the maximum decrease in the accumulation of photosynthetic attributes as compared to control conditions. However, it was noted that soil applied BC and PM treatments had a maximum impact on the improvement of chlorophyll and carotenoid content in tobacco leaves. The addition of individual application of PM improved the chlorophyll a (15.76 and 24.33%), chlorophyll b (40.00 and 42.03%), total chlorophyll (20.79 and 27.94%), and carotenoids content (42.27 and 56.44%) under control and drought stressed conditions, respectively. Similarly, sole application of BC improved the chlorophyll a (6.60 and 10.28%), chlorophyll b (22.51 and 25.89%), total chlorophyll (9.98 and 13.46%), and carotenoids content (30.70 and 36.32%) under control and drought stressed conditions, respectively. The combined approach of BC and PM treatment increased the content of chlorophyll a (14.45%), chlorophyll b (48.88%), total chlorophyll (21.32%), and carotenoids content (32.00%) respectively, compared to the control. More response in terms of maximum photosynthetic attributes was noticed where BC was applied at the rate 300 g kg^− 1^ of soil combined with the peatmoss, while minimum response was observed where no BC and peatmoss was applied under drought stressed conditions.

### Enzymatic antioxidants and ROS related attributes

Data depicted in Table 1 represented the impact of various levels of soil applied BC and mixed with the PM on the enzymatic antioxidant attributes of tobacco under drought stress in pot trials under greenhouse conditions. Drought stress increased the catalase activity (57.21%), superoxide dismutase activity (70.93%), peroxidase activity (54.35%), and APX activity (54.45%) in comparison with well-watered conditions. An optimal level of soil applied BC combined with PM decreased the enzymatic antioxidant in the tobacco leaves under drought conditions. The best level of BC treatment (300 g kg^− 1^) mixed with PM decreased the catalase activity (12.87 and 9.84%), superoxide dismutase activity (13.24 and 7.68%), peroxidase activity (16.29 and 7.51%), and APX activity (37.20%) under control and drought stressed conditions, respectively. The decreasing pattern in terms of enzymatic antioxidants and ROS attributes for biochar levels was control > 150 g kg^− 1^ > 300 g kg^− 1^.

**Table 1:** Interactive effect of various doses of soil applied biochar and peatmoss on the antioxidant enzymatic activities of tobacco grown under drought stressed conditions

Treatments			SOD (U g^− 1^)	POD (U g^− 1^)	CAT (U g^− 1^)	APX (U g^− 1^)
Control	PM (-)	BC_0_	518.77 ± 9.67 e	17088.89 ± 682.20 cd	522.10 ± 7.45 ef	0.41 ± 0.01 c
		BC_1_	490.52 ± 16.37 ef	16893.96 ± 253.16 cd	497.30 ± 10.74 fg	0.38 ± 0.01 c
		BC_2_	462.47 ± 8.99 f	15727.43 ± 439.91 d	460.55 ± 13.52 g	0.29 ± 0.02 d
	PM (+)	BC_0_	412.74 ± 13.88 g	13693.00 ± 915.37 e	389.53 ± 12.57 h	0.21 ± 0.02 e
		BC_1_	393.24 ± 14.82 gh	11579.41 ± 744.75 f	369.20 ± 13.74 hi	0.18 ± 0.01 ef
		BC_2_	345.66 ± 13.76 h	10038.17 ± 736.64 f	333.75 ± 15.13 i	0.14 ± 0.02 f
Drought Stress	PM (-)	BC_0_	873.72 ± 12.24 a	25521.19 ± 276.34 a	797.97 ± 10.98 a	0.53 ± 0.01 a
		BC_1_	832.69 ± 14.17 ab	24862.58 ± 408.91 a	769.93 ± 8.37 ab	0.49 ± 0.01 ab
		BC_2_	817.04 ± 15.07 b	23951.10 ± 392.25 a	726.87 ± 13.93 a	0.46 ± 0.01 b
	PM (+)	BC_0_	688.93 ± 6.11 c	19794.69 ± 405.01b	614.53 ± 16.43 c	0.38 ± 0.01 c
		BC_1_	646.29 ± 11.33 cd	19144.57 ± 332.32 b	583.08 ± 14.86 cd	0.33 ± 0.02 d
		BC_2_	626.17 ± 16.92 d	17959.13 ± 228.56 bc	551.61 ± 10.27 de	0.29 ± 0.02 d

For each parameter, bars with the same letter are not significantly different across treatment means (*p* ≤ 0.05) based on a Tukey’s-HSD test. Capped lines denote the standard deviation of three replicates. PM (+); with peatmoss; PM (-); without peatmoss; BC_0_ = no biochar; BC_1_ = 150 mg kg^− 1^; BC_2_ = 300 mg kg^− 1^

### Osmolytes and lipid peroxidation attributes

The tobacco plants subjected to drought showed a linear increase in the osmolytes attributes (Table 2). More accumulation of osmolyte attributes was noticed where drought stress was applied as compared to well-watered conditions. The best level of BC (300 g kg^− 1^) combined with the peatmoss (5%) decreased the proline and MDA content while improving the soluble sugar and soluble protein contents in tobacco plants in comparison with the control where no use of BC and PM was carried out. The optimal level of biochar in combination with and without peatmoss showed an increase in the soluble sugars (17.65 and 12.20%), and soluble protein (31.16 and 15.88%) and a decrease in the leaf proline accumulation (13.92 and 9.03%) and MDA content (16.40 and 8.65%) under control and drought stressed conditions, respectively.

**Table 2:** Interactive effect of various doses of soil applied biochar and peatmoss on the osmolytes and lipid peroxidation activities of tobacco grown under drought stressed conditions

Treatments			Soluble Protein (mg g^− 1^)	Soluble Sugar (mg g^− 1^)	Proline (µg g^− 1^)	MDA (nmol g^− 1^)
Control	PM (-)	BC_0_	25.43 ± 3.29 g	32.22 ± 3.40 h	49.59 ± 1.32 ef	46.95 ± 1.29 d-f
		BC_1_	33.70 ± 1.26 f	36.04 ± 1.79 h	47.03 ± 1.22 ef	42.45 ± 1.52 e-g
		BC_2_	38.77 ± 1.62 ef	38.99 ± 1.87 gh	45.74 ± 1.76 fg	40.36 ± 1.72 f-h
	PM (+)	BC_0_	40.09 ± 1.19 ef	44.15 ± 2.15 fg	39.78 ± 1.88 gh	38.51 ± 1.76 gh
		BC_1_	45.52 ± 0.86 de	48.85 ± 1.96 f	35.54 ± 1.78 hi	34.92 ± 1.85 hi
		BC_2_	47.17 ± 1.18 d	50.85 ± 2.04 ef	31.18 ± 2.67 i	31.07 ± 1.72 i
Drought Stress	PM (-)	BC_0_	51.74 ± 2.04 cd	56.77 ± 2.73 de	67.51 ± 1.30 a	61.70 ± 1.26 a
		BC_1_	55.78 ± 2.20 c	59.19 ± 1.94 d	65.75 ± 2.22 ab	58.85 ± 2.41 ab
		BC_2_	58.08 ± 2.38 c	64.49 ± 1.72 cd	63.67 ± 1.01 ab	54.52 ± 1.45 bc
	PM (+)	BC_0_	66.01 ± 1.25 b	69.74 ± 1.60 bc	60.26 ± 1.02 bc	50.07 ± 1.69 cd
		BC_1_	70.63 ± 1.60 b	73.07 ± 1.38 ab	55.73 ± 0.48 cd	51.89 ± 2.32 cd
		BC_2_	78.37 ± 2.32 a	77.48 ± 2.32 a	52.56 ± 2.01 de	47.63 ± 2.46 de

For each parameter, bars with the same letter are not significantly different across treatment means (*p* ≤ 0.05) based on a Tukey’s-HSD test. Capped lines denote the standard deviation of three replicates. PM (+); with peatmoss; PM (-); without peatmoss; BC_0_ = no biochar; BC_1_ = 150 mg kg^− 1^; BC_2_ = 300 mg kg^− 1^

### Leaf quality attributes

The addition of various levels of soil-applied BC combined with the PM caused significant change (*p* ≤ 0.05) in leaf quality attributes of tobacco plants (Table 3). Change in the quality attributes was noticed by the induction of drought stress. Drought-stressed conditions decreased the leaf K^+^ (14.57%) and increased the leaf Cl^−^ (8.60%) and leaf nicotine (23.48%). The best level of BC (300 g kg^− 1^) combined with the peatmoss (5%) caused a significant change in the quality attributes of the tobacco grown under drought and control conditions. More response in terms of maximum leaf quality attributes for improved nicotine was noticed where BC was applied at the rate 300 g kg^− 1^ of soil combined with the peatmoss, while minimum response was observed where no BC and peatmoss was applied under drought stressed conditions.

**Table 3:** Interactive effect of various doses of soil applied biochar and peatmoss on the osmolytes and lipid peroxidation activities of tobacco grown under drought stressed conditions

Treatments			Leaf K^+^ (mg g^− 1^)	Leaf Cl^−^ (mmol/g)	Leaf Nicotine (mg g^− 1^)
Control	PM (-)	BC_0_	9.41 ± 0.31 ef	0.11 ± 0.008 a-c	5.73 ± 0.16 b
		BC_1_	9.88 ± 0.64 d-f	0.10 ± 0.017 b-d	5.26 ± 0.09 c
		BC_2_	10.87 ± 0.42 c-e	0.09 ± 0.011 c-f	4.45 ± 0.17 d
	PM (+)	BC_0_	12.80 ± 0.50 ab	0.10 ± 0.008 b-e	3.62 ± 0.11 e
		BC_1_	13.13 ± 0.55 a	0.08 ± 0.008 c-f	3.27 ± 0.07 ef
		BC_2_	13.76 ± 0.37 a	0.07 ± 0.005 ef	3.14 ± 0.04 f
Drought Stress	PM (-)	BC_0_	7.21 ± 0.76 g	0.14 ± 0.008 a	6.40 ± 0.05 a
		BC_1_	8.81 ± 0.50 fg	0.12 ± 0.008 ab	6.16 ± 0.09 a
		BC_2_	9.37 ± 0.25 ef	0.11 ± 0.012 a-c	6.05 ± 0.09 ab
	PM (+)	BC_0_	10.87 ± 0.42 g	0.10 ± 0.008 b-e	4.45 ± 0.17 d
		BC_1_	11.26 ± 0.10 fg	0.07 ± 0.008 d-f	4.32 ± 0.06 d
		BC_2_	12.14 ± 0.27 ef	0.06 ± 0.009 f	4.07 ± 0.15 d

For each parameter, bars with the same letter are not significantly different across treatment means (*p* ≤ 0.05) based on a Tukey’s-HSD test. Capped lines denote the standard deviation of three replicates. PM (+); with peatmoss; PM (-); without peatmoss; BC_0_ = no biochar; BC_1_ = 150 mg kg^− 1^; BC_2_ = 300 mg kg^− 1^

### Correlation matrix

**Fig. 4:**
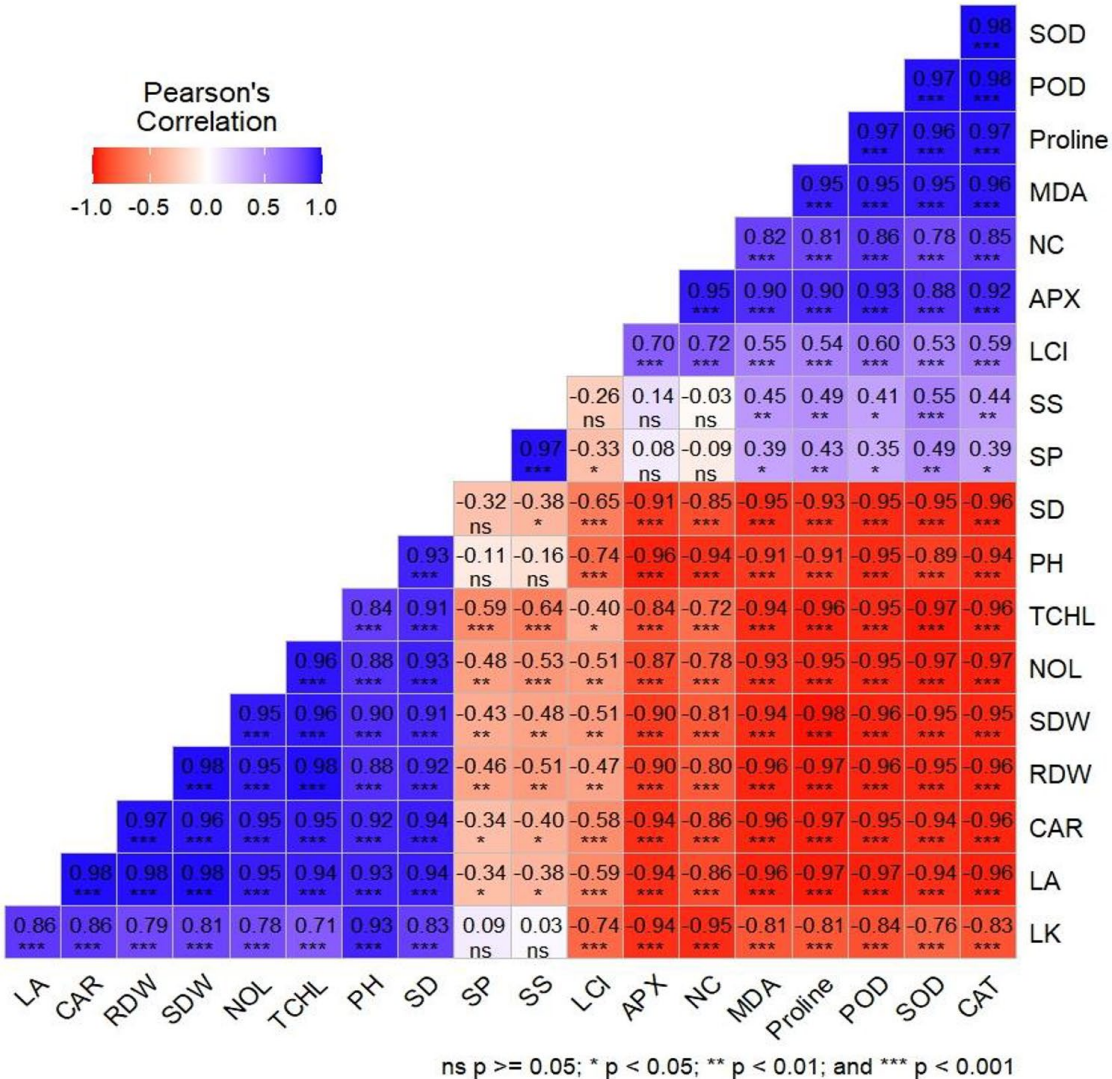
Correlation analysis of measured parameters of tobacco plants under various levels of soil applied biochar and peatmoss (with and without) and drought stress in pot trial experiment. NOL = number of leaves; LL = leaf length; LW = leaf width; PH = plant height; LA = leaf area; SFW = shoot fresh weight; SDW = shoot dry weight; RFW = root fresh weight; RDW = Root dry weight; MDA = malonaldehyde content; PRO = proline content; SP = soluble proteins; TCHL = total chlorophyll content; SOD = superoxide dismutase activity; CAT = catalase activity; POD = peroxidase activity; SS = soluble sugar content; APX = ascorbate activity; LK = leaf K content; L Cl = leaf chloride content; NC = nicotine content

A clear association was evident among all growth, biochemical, lipid peroxidation, enzymatic, and quality related variables of tobacco plants. Photosynthetic attributes like chlorophyll and carotenoid content exhibited negative correlations (*p* < 0.001) with enzymatic activities, MDA, and proline accumulation. Proline and MDA displayed significant negative associations (*p* < 0.001) with total chlorophyll, carotenoid content, plant height, biomass (fresh and dry), and quality related attributes of tobacco. Similarly, photosynthetic attributes exhibited significant positive correlations (*p* < 0.001) with growth and biomass (fresh and dry) characteristics of tobacco (Fig. 4).

### Principle component analysis

**Fig. 5:**
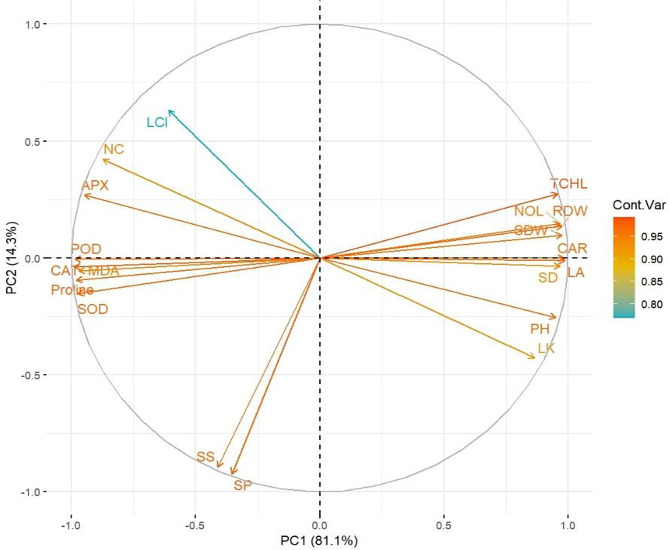
Principal component analysis plot showing loadings of measured parameters and contribution of two principal components (PC1 and PC2), under various levels of soil applied biochar and peatmoss (with and without) and drought stress in pot trial experiment. NOL = number of leaves; LL = leaf length; LW = leaf width; PH = plant height; LA = leaf area; SFW = shoot fresh weight; SDW = shoot dry weight; RFW = root fresh weight; RDW = Root dry weight; MDA = malonaldehyde content; PRO = proline content; SP = soluble proteins; TCHL = total chlorophyll content; SOD = superoxide dismutase activity; CAT = catalase activity; POD = peroxidase activity; SS = soluble sugar contents; APX = ascorbate activity; LK = leaf K content; L Cl = leaf chloride content; NC = nicotine content

Principal component analysis revealed two major clustering among others accounting for 95.4% variability of PC1 and PC2 components (Fig. 5). One of the clusters consists of POD, SOD, Proline, MDA, and other measured parameters which showed associations when subjected to drought stress, peat moss, and various levels of soil applied biochar applications. This group is plotted in the negative PC1 and PC2 axes and is dominated by experimental treatments without peat moss and drought conditions. The other cluster consists of a range of measured parameters such as total chlorophyll contents, root dry weight, number of leaves and carotenoid content, and others and plots on the positive PC1 axis. This group is characterized by the application of biochar and peat moss applications as part of tobacco plant treatments. Both soluble sugars and soluble proteins contents showed significant association among themselves by plots away from the major clusters but did not exhibit any noticeable association with them.

### Chord analysis

**Fig. 6:**
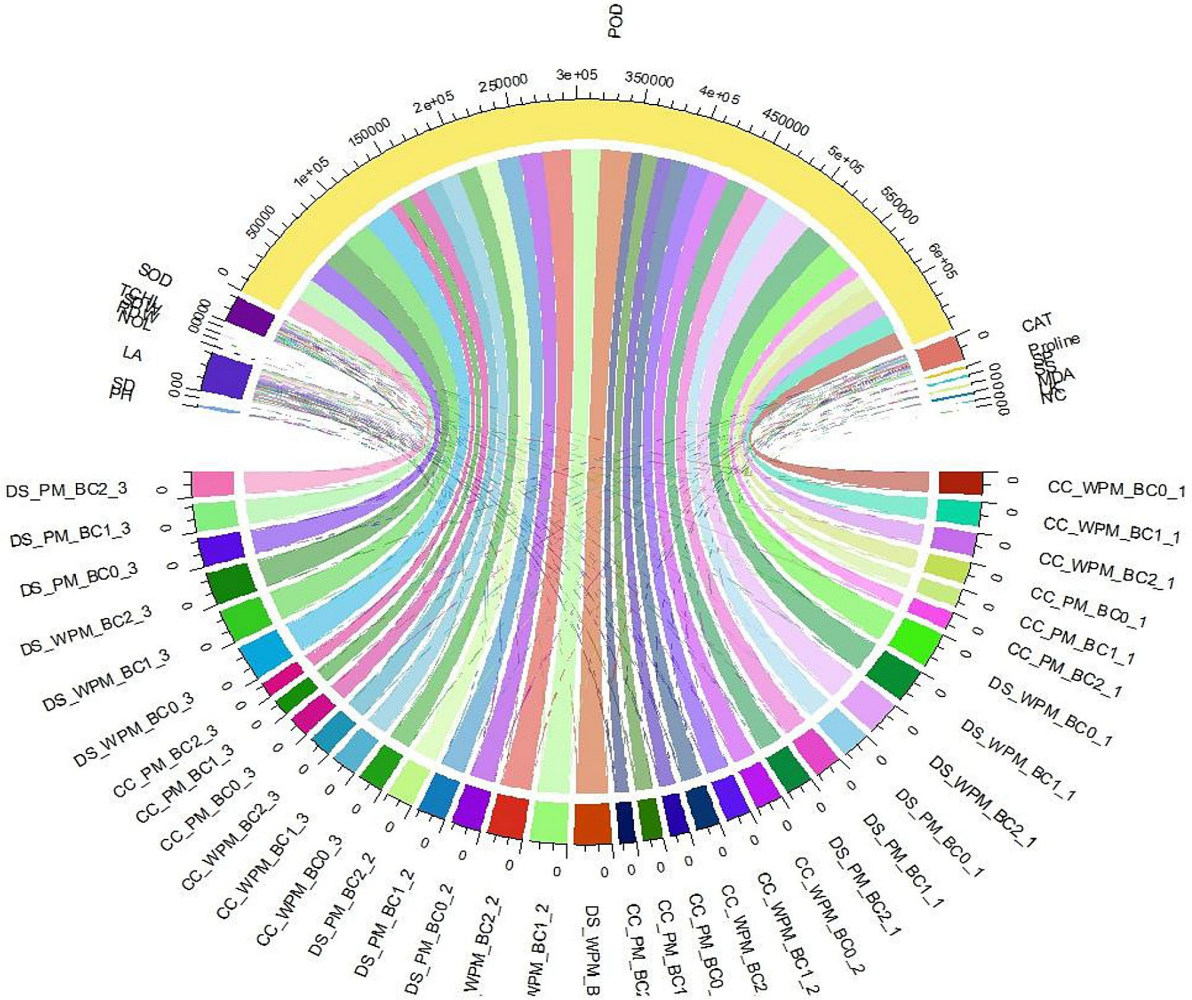
Chord diagram depicting the association and contribution of each measured parameter of tobacco plants under various levels of soil applied biochar and peatmoss (with and without) and drought stress in pot trial experiment. NOL = number of leaves; LL = leaf length; LW = leaf width; PH = plant height; LA = leaf area; SFW = shoot fresh weight; SDW = shoot dry weight; RFW = root fresh weight; RDW = Root dry weight; MDA = malonaldehyde content; PRO = proline content; SP = soluble proteins; TCHL = total chlorophyll content; SOD = superoxide dismutase activity; CAT = catalase activity; POD = peroxidase activity; SS = soluble sugar content; APX = ascorbate activity; LK = leaf K content; L Cl = leaf chloride content; NC = nicotine content

The chord diagram (Fig. 6) showed flows among measured variables and entities of tobacco plant experiment subjected to stress conditions and the applications of biochar and peat moss. Bar thickness reflects the degree of variation in each category, and the direction of the lines points to a relationship between categories. The peroxidase activity, leaf area, superoxide dismutase activity, and catalase activity dominated the flows and associations among the measured variables helping understand the crucial directional relationships and allowing insights into the data set. The drought stress conditions (indicated by the abbreviation DS) and related entities are shown to relate to peroxidase activity by the curved lines.

## Discussion

Plants under drought stress may have less water accessible to them, which might impede their growth and lower food production [36].

The present investigation demonstrated that tobacco plants cultivated under drought conditions showed enhanced growth, biomass, chlorophyll content, and antioxidant activity, which were contingent upon the quantity of soil amendments supplied. By limiting the absorption of chloride ions, this improvement not only enhanced the qualitative traits but also improved growth and biomass. Effective mitigation of the adverse impacts of water-deficit-induced stress in tobacco plants can be achieved by optimum use of peatmoss and rice straw biochar.

Based on the data from this study, it was found that tobacco plants experienced a significant decrease in vegetative development and biomass growth when exposed to different levels of water stress compared to well-watered conditions. Previous research indicates that there is a direct correlation between drought and the decline in growth and biomass attributes in tobacco and other field crops [37, 38]. Under control conditions, such enhancements can be attributed to enhanced physiological processes, increased nutrient absorption, and maximum photosynthetic attributes [39]. This can lead to an increase in the number of leaves, larger leaf areas, and overall improved plant growth and yields. On the other hand, the stress caused by changes in water levels negatively impacts the growth and yield of crops [40]. This stress disrupts the normal functioning of physiological mechanisms, causing damage to cell membranes and photosystems (Figs. 1, 2 and 3). The present investigation revealed that the combined application of PM and BC to drought-stressed plants resulted in an increase in leaf area, enhanced growth, and a higher percentage of fresh and dry biomass [41, 42]. It was noted that the presence of the required essential nutrients in the rhizosphere by the PM and BC leads to an increase in cell division, resulting in improved plant height and leaf growth [43]. Additionally, the application of PM and BC to the soil helps mitigate the harmful effects of drought stress on plant cells [44, 45]. The enhancement can be ascribed to the biochar’s extensive surface area and its ability to improve soil health, resulting in increased water absorption and mitigating the negative effects of water scarcity on plant growth and development. Different kinds of field crops showed similar results in terms of increased plant growth and biomass when exposed to drought stress and treated with BC and PM [46, 47]. It was noticed that when BC and PM are directly applied, they function as regulatory chemicals that supply the necessary nutrients to stimulate plant cell division and cell elongation in the presence of abiotic stress [48, 49].

Drought stress is a significant factor leading to crop loss, primarily due to inhibition in the photosynthetic process [50]. Soil supplements, PM, and BC have significant ability to improve the process of photosynthesis in plants [51, 52]. This area of study has been extensively studied as a viable approach to alleviate the adverse impacts of drought stress on plants. Soil amendments have been shown to have beneficial effects on the photosynthetic attributes of tobacco plants in drought conditions, particularly in terms of plant vigor, growth, and biomass [53]. The combined use of both biological stimulant compounds, BC and PM, significantly improved the levels of chlorophyll and carotenoids in plants subjected to drought stress. Usually, drought stress can decrease the chlorophyll levels in plants, which hinders the process of photosynthesis [54]. Research has demonstrated that the use of soil amendments can increase the chlorophyll levels in various field crops [55]. Our investigation revealed that the combined use of soil amendments effectively increased the chlorophyll content of tobacco plants (Fig. 3) under drought conditions. Furthermore, our findings confirm previous studies and demonstrate that the application of BC and PM can mitigate the negative effects of drought-induced stress on chlorophyll and carotenoid levels [56].

Drought is among the major stressors that can irreversibly damage plants’ cellular functions and structures including membrane lipids, proteins, and DNA, which is most commonly caused by the production and accumulation of reactive oxygen species (ROS) [57]. Levels of MDA are widely reported to evaluate the generation of ROS accumulation in plants [58]. Significantly elevated levels of enzymatic antioxidants and MDA were observed in tobacco plants under drought stress. To counteract this increase, plants established mechanisms to inhibit the accumulation of reactive oxygen species (ROS) that damage cellular stability. The mechanisms encompass the storage of specific categories of osmoprotectants referred to as compatible solutes [59]. These solutes include total soluble sugars and proteins which actively play a role in plants’ ability to combat drought stress induced damage [60]. Their contribution is mostly involved in maintaining water balance in plants and turgor levels and assisting in maintaining overall physiological attributes [61]. Additionally, the accumulation of osmolytes in plant tissues can play a role as antioxidants to scavenge ROS [62]. Furthermore, results point to the fact that the use of soil amendments enhances the contents of these compounds under drought stress. The role of BC and PM in increasing the capacity of tobacco plants to resist and tolerate drought stress is in accordance with previous studies [63, 64]. Meanwhile, water-stress-resistant plants can also respond well in adapting to water stress by changing their cellular system and activating various internal defensive mechanisms, including the activation of antioxidant enzymes [65]. Accordingly, well boosted antioxidant metabolism in plants can contribute to a plant’s capability to scavenge ROS. Overall, the results revealed that combining soil amendment contributed to enhanced antioxidant enzymes in plants [66].

In most plants, the sugar content decreases in response to abiotic stress [67]. Both under normal and drought-stressed conditions, the application of sustainable soil amendments promotes the integrating, movement, and absorption of carbohydrates. As a result, the plant has increased availability of energy in the form of free sugar molecules. This finding has been confirmed by the data indicating a more significant variation in fresh weight and improvement in the initial stages of growth and development in crops (Figs. 1 and 2). We have concluded that this rise in available energy and osmolytes by measuring the total free sugar contents under control conditions. Increased sugar availability would serve as a significant advantage when plants are exposed to stress conditions and possibly explain the fact that most stress response indicators such as chlorophyll contents (Fig. 3), proline content (Table 2), and increased potassium (Table 3) are far less in drought stress conditions but moderately higher where the combined application of BC and PM was done. These data demonstrate that the tobacco plants applied with the BC and PM experienced less stress, most likely due to the enhanced sugar availability that in turn enables an effective stress response. The correlation analysis clearly depicts how effectively the synergistic effects of these treatments among the mitigate drought-induced damage, thereby providing insights into the most impactful variables contributing to improved plant resilience (Fig. 4).

Tobacco is a significant cash crop, with its economic significance tied to the growth of leaves, and the accumulation of nicotine in its leaves. Nicotine is synthesized through the ornithine and arginine pathways in root cells and then transported to the leaves through the xylem. In the leaves, nicotine is stored in the vacuole [68]. In addition, the process of producing and storing nicotine in plants, known as biosynthesis and accumulation, can be influenced by a range of abiotic stresses [69]. The application of soil amendments in this study significantly increased the buildup of nicotine in the leaves of flue-cured tobacco (Table 3). These findings can be linked to previous research that has shown that the application of soil amendments improves the absorption, accumulation, assimilation, and metabolism of nitrogen, which ultimately results in the improved quality of field crops [70].

## Conclusion

In conclusion, the current findings showed that drought stress negatively affects tobacco plants by disrupting major physio-biochemical and quality attributes. Drought raised the concentration of lipid peroxidation and proline accumulation and limited the overall quality attributes of tobacco plants. The synergistic approach of biochar and peatmoss significantly decreased the drought-induced reductions in tobacco plant development by improving nutrient supply and uptake potassium, enhancing photosynthetic capacity, strengthening the antioxidant system (increasing antioxidant activity), and promoting osmolyte accumulation. Findings depicted that using biochar and peatmoss is an efficient ecological and sustainable approach to increase tobacco growth under drought conditions, making them valuable tools for promoting plant growth in water-scarce environments.

## Data Availability

The datasets analyzed during this study are included in this manuscript.

## **Declarations**

### Statement on guidelines

All experimental studies and experimental materials involved in this research are in full compliance with relevant institutional, national, and international guidelines and legislation.
